# Can Nursing Drive Technological Advances in Healthcare in the Asia-Pacific?

**DOI:** 10.31372/20180304.1022

**Published:** 2018

**Authors:** Joseph Andrew T. Pepito, Rozzano C. Locsin

**Affiliations:** a University of the Visayas, Cebu, Philippines; b Tokushima University, Tokushima, Japan

**Keywords:** nursing, technological advances, human caring, biodesign, challenges of technology

## Abstract

The Asia-Pacific healthcare industry is expected to grow at 11.1% in 2018. This has been considered one of the fastest growing regions in the world. The positive growth occurring in the Asia-Pacific region is due to the increasing adoption of technology. While it is understood that technology drives advances in nursing and the health sciences, would it be possible that nursing can or will also drive technological advancements in human caring? All too often, nurses are employed in health care as simply the end-users of technologies. It is the purpose of this paper to engage a discourse towards advancing nursing as driving technological improvements aimed for human caring. How can nursing facilitate this powerful dynamic, and what will it take for nursing as a discipline and a profession to occupy a primary role in this all too often unrecognized view, that nursing can and will drive technological advancements for human caring?

## Introduction

The Asia-Pacific region is comprised of 48 countries and accounts for about 60% of the world’s population despite only possessing 21% of the earth’s land mass. It includes most of South Asia, East Asia, Southeast Asia, and Oceania. The Asia-Pacific healthcare industry is expected to grow at 11.1% to reach US$ 517 billion in 2018. This has been considered one of the fastest growing regions in the world (Frost & Sullivan, 2018). The positive growth occurring in the Asia-Pacific region is due to the increasing adoption of technology, innovative healthcare access programs, and delivery of care that is not within the traditional hospital settings.

The Asia-Pacific region has consistently featured in World Health Organization (WHO) analyses of critical shortages of health workers over the last decade (Yeates & Pillinger, 2018). It is therefore critical for stakeholders, especially nurses, to come together and share best practices and develop innovations that are able to meet the needs of patients from both developed and developing nations. Southeast Asia accounts for three-quarters of all healthcare worker shortages globally.

To address the problem of nursing shortages, the American Organization of Nurse Executives (AONE) made specific recommendations to investigate the use of technology to enhance the capacity of a reduced nursing workforce (Kennedy, 2003). Many advances in technology have been made available to help nurses in the performance of their jobs within the past decade. These advances have enabled them to care for patients more efficiently and safely (Thimbleby, 2013). Nursing is not the same as it was thirty years ago due to the adoption of technology in nursing practice.

For the region, challenges in healthcare access and the affordability of new technology demand complex and innovative solutions from healthcare industry stakeholders. Frost and Sullivan (2018) identified key issues in the industry that warrant attention for the development of patient-focused technology platforms. One of the major challenges of nursing education and nursing practice today is the demand inherent in technological innovations in health care. Therefore, if nursing is to remain relevant in a technological world (Pepito & Locsin, 2018), practice processes which are responsive to disciplinary and professional obligations, particularly the mandate for caring in the human health experience (Newman, Sime, & Corcoran-Perry, 1991), are a requisite. Nursing and the images of current nursing practices seem to be moving towards a revolution in nursing science in order to adopt a responsive view of a technologically advanced nursing care world. With that in mind, a question that can be asked is, “can nursing drive technological advances in human care in the Asia-Pacific?”

## Driving Technological Advances in Human Caring

One of the major challenges of nursing education and nursing practice today is the demand inherent in technological innovations in health care. Technological competency in nursing practice is not new. It is a practice of nursing focused on nurses’ proficiency with skills. Certainly, continuing the practice of nursing as task completion does not demonstrate progress towards a future that meets the demands of human care in a technological world. Innovations, adaptation, and adoption are the keys to an engaging professional practice of nursing. One of these innovative technological designs is Biodesign.

## Technology Development through Biodesign

The Biodesign innovation process is a series of structured procedures that result in the development of new medical technologies. This process was developed by the Stanford University’s “Biodesign” Program, which has served as the foundation for the development of medical technologies (Schwartz, Kumar, Azagury, Brinton, & Yock, 2016). Biodesign is a process designed to analyze validated clinical needs which are unmet and utilizes a structured filtering process to find the most significant and compelling needs that merit solving. The Biodesign process takes into consideration the stakeholder’s involved, business models, regulatory pathways and strategies for reimbursements, and creative solutions for the prioritized needs that were determined by multidisciplinary teams.

The Biodesign process consists of three phases, (1) Identify, (2) Invent, and (3) Implement. The Biodesign process is both cyclical and iterative and often involves returning to earlier stages and phases as new information becomes available through research.

Phase I: Identify (Clinical Immersion, Needs Finding)

The first phase of the Biodesign process involves intensive needs finding. After the identification of needs, the development of a need statement occurs next. This is a single sentence that is carefully crafted to capture the essence of the need. It outlines the clinical problem that was identified, the specific population that has been affected by the problem, and a measurable result that can be affected by possible solutions.

Phase 2: Invent (Concept Generation and Screening, Creativity)

The second phase involves brainstorming for solutions (concepts) that can potentially meet the need criteria. It is in this phase where input from experts is essential. The results of the brainstorming will be filtered to ensure that the concepts will truly meet the need criteria. Criteria for filtering include intellectual property (IP), regulatory pathway, reimbursement potential, technical feasibility, and viability of the model for business in order to bring the solution to the population in need. After the filtering of concepts, proto-typing occurs.

**Figure 1:**
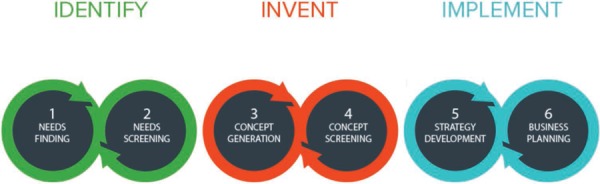
The three phases of Biodesign. Stanford Byers Center for Biodesign, 2018.

Phase 3: Implement (Commercialization Potential, Strategy Development)

The third phase involves an in-depth analysis of each possible solution in order to create a plan to proceed. Included in this analysis is a comprehensive understanding of the IP landscape, plans for a credible reimbursement pathway, an understanding of the engineering feasibility, resources, and personnel that are required for undertaking further research and development. After which, a detailed plan for device testing is constructed, which involves pre-clinical, clinical trials, and quality management protocols. A viable business model would then be created (Zenios et al., 2010).

Frontline nurses and other multidisciplinary healthcare team members stressed the importance of getting those engaged in direct patient care involved in the design, selection, and the testing of the technology. These are steps that are often overlooked when new systems or devices are acquired (Hamer, 2013).

In the 1^st^ phase of the Biodesign process, the needs of the industry are identified. It is at this phase where input from nurses may help determine which aspects of their profession require improvements that can be addressed by technology. In the 2^nd^ phase of the Biodesign process, input from the nurses during the brainstorming session will be very useful in developing technologies catering to the needs of patients while ensuring that the technology will enable nurses to provide a more holistic type of care.

An improvement in healthcare quality is the likely benefit of technology but another point of consideration is concerned with the disadvantages that result in work errors arising from the use of new technologies. The benefits of technology may not be realized mainly due to four reasons (Jelec, Sukalic, & Friganovic, 2016):

1. Non-observance of human factors and ergonomic principles leading to bad technological design;
2. A technological interface that was not designed for the patient or the environment;
3. Inadequate plans for the implementation of new technologies into practice;
4. Maintenance plans that are inadequate.

## How Technology Has Changed Nursing

In the late 1960s, the best tool of a nurse was pen and paper. Today, many nurses use a computer to complete their patient care management documentation. Prior to the development of medical technology, nurses relied on their own senses to monitor patient care status and for the detection of specific physiological changes. Today, there are a host of medical technologies that monitor a patient’s status. New ways of documenting and monitoring a patient’s status and use of telehealth technologies allow patients to receive care in their preferred locations are just some ways that technological advances are changing how nurses perform their care duties.

A front runner in technological innovations in the Asia-Pacific region is Singapore (Tao, 2017). An example of Singapore’s innovations in healthcare technology is their “Hospital to Home” program. This program aims to reduce the risk of re-admission of patients who have multiple medical conditions by providing help in managing their transition to home care. Care coordinators are employed by Sing Health institutions and are taught how to conduct follow-ups through the use of information technology, connected care technologies such as Telehealth, and home visits that ensure patients and their caregivers are able to implement the discharge care plan that has been developed by a team of “patient navigators, ” who are registered nurses.

Another program that has been implemented in Singapore is called the Acute Medical Ward (AMW). The AMW uses technology in the form of a virtual ward to cater to patients awaiting beds but are still located in the Emergency Medicine Department (EMD). The patients under AMW care are able to receive diagnostic tests, procedures, and medications similar to services provided in general wards despite being physically still at the EMD through the use of virtual wards (National University Hospital, 2015). This redesign of workflow and process has reduced the average length of hospital stay by 2.4 days, and has decreased the average patient bill by 20% (Balakrishnan et al., 2017).

Singapore has also implemented the National Electronic Health Record (NEHR); its purpose is to have a single electronic health record per patient. This would enable healthcare providers, such as nurses, to access details of their patient care plans and histories prior to deciding on treatments, even if patients move from one hospital to another. Due to NEHR’s implementation, Singapore continues in its aims at improving patient experience, increasing productivity in healthcare institutions, and promoting interoperability among disparate health information systems.

Japan is also at the forefront of healthcare technology and robotics in the Asia-Pacific region. In addition to the adoption of healthcare informatics technology, Japan has also developed robotic technology for healthcare. A robot system for walking support dubbed “Robot Suit HAL” is designed to assist a person in walking. The nursing care assistant robot named “RIBA” is designed to aid in moving a person from their bed. “Paro” is another robot that has been developed to aid in the treatment of mental health for older adults (Shimizu, 2017; The Economist, 2017). These robotic technologies are already performing some of the functions of nurses which have an impact on the practice of nursing. An attempt to explore the critical role of humanoid robots in replacing human nurses was also examined by Locsin and Ito (2018).

Unlike Singapore and Japan, most of the other countries in the Asia-Pacific region are considered developing countries. Countries such as Bangladesh, China, India, Indonesia, Malaysia, the Philippines, Thailand, Vietnam, Fiji, Palau, Papua New Guinea, Samoa, and Vanuatu (Department of Foreign Affairs (Australia), 2015) are challenged by severe limitations of resources. Development of modern healthcare services that deliver sensible medical technologies is a great challenge due to the lack of expertise in technological development, which forces developing countries to import technology in healthcare (Banta, 1986).

The main benefit of technology in nursing is the improvement of patient care. It improves the effectiveness and efficiency by which care is delivered (Bowles, Dykes, & Demiris, 2015). Much of the technology being implemented in nursing practice is geared towards the improvement of nurses’ communication with other healthcare providers. Technology in nursing improves documentation in healthcare (Rouleau et al., 2017). With the use of technology, nurses can deliver a better patient experience with the use of electronic charts that can efficiently record and store a patient’s files (Palabindala, Pamarthy, & Jonnalagadda, 2016). Patients can also get access to their own medical records and test results through the use of online patient portals (Griffin, Skinner, Thornhill, & Weinberger, 2016). Communication with the nurse, doctors, and other members of the healthcare team is improved through the use of phones and pagers (Wu et al., 2011). GPS tagging and tracking of equipment (Kamel Boulos & Berry, 2012), improved diagnostic devices (Balogh et al., 2015), and smarter alarm systems (Bach, Berglund, & Turk, 2018) all contribute to the delivery of an improved patient experience through the use of technology. The use of technology as Elrick (2017) proposed also contributes to better outcomes in healthcare and assists nurses in significantly improving customer satisfaction and engagement.

A potential drawback with using technology in nursing is the learning curve, when a novel technology is implemented (Zadvinskis, Garvey Smith, & Yen, 2018). A decreasing level of effectiveness in communication through the use of technology has also been observed (Rouleau et al., 2017). For example, during electronic charting, the application used does not possess adequate options to allow nurses to properly convey information. Not being able to communicate properly through applications results in frustration of healthcare providers. There are risks in privacy through the use of technologies which can potentially result in breaches in patient confidentiality (Malin, El Emam, & O’Keefe, 2013). Similarly, Weiner and Biondich (2006) argue that technology is also affecting nurses’ interactions with patients.

The difficulties experienced with the adoption of medical technology in developing countries, particularly in the Asia-Pacific region, is exacerbated by a partial understanding of the cultural, social, economic, and institutional factors that affect technology development, transfer, dissemination, and use (Bonair, Rosenfield, & Tengvald, 1989). Furthermore, in developing countries in the Asia-Pacific Region, there are added challenges in the adoption of innovative healthcare technologies. The technology may not be suitable to local conditions and its use may be limited due to lack of expertise. Affordability is also an issue in developing countries in the Asia-Pacific region due to the limited resources to be invested in technological development (Bonair et al., 1989). Kathuria (1987) has declared that the added challenge in the adoption of healthcare technology in developing countries is that imported technology from developed nations has not been designed for use in developing nations. There is concern regarding difficulty in the assimilation and adaptation of imported technologies to local conditions. Table 1 provides examples of emerging technologies that are changing the way nursing is practiced and the challenges that are faced by nurses (Huston, 2013).

**Table 1:** Examples of Emerging Technologies That Are Changing Nursing Practice

Technology	Benefits	Challenges
Genetics and Genomics	Most of the disease risks, health conditions, and therapies used to treat these conditions have been found to have a genetic and/or genomic element that is influenced by environmental, life-style, and a host of other factors that influence the entire nursing profession.	Majority of the nurses that are currently in practice are uninformed about genetics and genomics and this results in a lack of competence needed to effectively counsel and educate patients.
Tools for Diagnostics and Treatment that are less invasive and more accurate	Tools for diagnostics and treatment that are non-invasive and minimally invasive generally results in lower patient risk and cost.	The rate of introduction of new noninvasive and minimally invasive tools is a challenge for nurses and may result in a lack of competency.
3-D Printing	Bioprinters that utilize living cell mixtures can build a 3D structure of cells, layer by layer, in order to form human tissue and may eventually provide a replacement for human organs.	The healthcare industry is just beginning to explore the capabilities of the technology. There are limitations to the type of materials which can be used for printing.
Robotics	Improved diagnostic abilities, less invasive and more comfortable experience for patients, and the ability to carry out smaller and more precise interventions may be made possible through robotics. Robots can be used as adjunct care providers for some physical and mental health care provision.	More research is needed on comparing the effectiveness between robots and human healthcare providers. Concern has been expressed by healthcare providers about the lack of emotion in robots, suggesting that robots will never replace altogether, human healthcare providers.
Biometrics	Biometrics improves the security of confidential healthcare information and eliminates the costs of managing passwords that can be lost.	More research is needed in terms of cost, accuracy, and potential users’ resistance to change.
Electronic Healthcare Records (EHR)	Critical patient information can be accessed by multiple healthcare providers at all times which allows for better coordinated care.	Cost of implementation, enabling computers to communicate with each other and the debate on who “owns” the data in an EHR challenges its required implementation.
Computerized Physician/Provider Order; Entry (CPOE) and Clinical Decision Support	CPOE and Clinical Decision Support would fundamentally change the ordering process which may result in lower costs, reduced medical errors, and more interventions that are based on evidence and best practices.	The introduction of CPOE and clinical decision support requires altering the practice of healthcare providers. Resistance may be common due to the time spent on order entry. The implementation and training costs may be significant.

## Challenges in Nursing Practice with Technologies of Health care

While technologies of health care are challenging nursing practices, it is critical for nursing to advance its imagination, innovation, and creativity. Identified in Table 2 are the four leading challenges that nurses distinctly need to address as integral to nursing practice.

**Table 2:** The Four Leading Challenges for Nurses in Integrating Technologies in their Practice

Challenges	Description
Balancing human element with technology	Nurses must ensure that the human element is not lost in the expansion of technology. The human connection is the art of nursing and nurses must ensure that technology is used to supplement and not eliminate human resources.
Balancing costs and benefits	Cost is a challenge of a healthcare system that is driven by technology.
Training a technologically enabled nursing workforce and assuring ongoing competency	Nursing is an information-based profession and it is technology that enables nurses to bring that technology to the point of care. With rapid emergence of technologies, who will train healthcare professionals that work with new emerging technologies? Who will assure ongoing competency in an era where technologies can be obsolete in three years or less?
Ensuring that the use of technology is in accordance with the code of ethics.	‘How’ and ‘why’ technology should be implemented and what parameters should be put in place to determine its ethical use?

## The Conundrum of Caring in Nursing with Technologies in the Asia-Pacific

Due to the prominence of machine and medical technologies, nursing care is frequently based on procedures that aid in prolonging patients’ lives, but do not adequately meet their need for care (Locsin, 1995). Many patient encounters with nurses revolve around complex equipment and technologies in healthcare which are monitored and documented by nurses. Contemporary concerns about patient care in nursing are that there is too much dependence upon the expert utilization of technology and that nursing routines have become machine-oriented to a high degree (Locsin, 1995).

One of the qualities that constitute our human nature is compassion. It is a fundamental human attribute that people demonstrate towards other people (Boykin & Dunphy, 2002). It is the act of altruism or feeling of concern towards another human being (Beare & Myers, 1990). Some nursing proponents fear that compassion and caring are no longer the central theme to the nursing profession and has become a secondary concern to the laborious and overworked nurse in a setting that is technologically dominated (Adams, 2016).

## Caring in Nursing

Caring is the central expression of nursing (Boykin & Schoenhofer, 2001). Professional nurses are pressured to achieve greater technological proficiency and this has caused a re-thinking of the technology-caring dichotomy. The emphasis on technologic competency for exceptional nursing practice is considered essential to the contemporary practice of nursing. In caring for patients in an increasingly technological world, nurses recognize that technological proficiency is an advantage and an enhancement for caring but is not a substitute. In technologically demanding environments, competence with technology and equipment is seen as the principal expression of nursing as caring while technologic incompetence is equivalent to not caring (Locsin, 1995).

Caring is the reciprocal relationship between persons being cared for and those caring for them, in which the ultimate purpose is to foster compassion and growth in caring (Boykin & Schoenhofer, 2001), Nursing is the engagement of the nurse and the patient for the purpose of appreciating patients as participants in their own care, rather than as objects of healthcare. In fosterning this engagement, patients and nurses may live their own lives more meaningfully.

## The Theory of Technological Competency as Caring in Nursing

*Technological Competency as Caring in Nursing* (Locsin, 2005) is a middle range theory founded in the general theory of *Nursing as Carin*g by Boykin and Schoenhofer (2001). This theory illustrates nursing practice grounded in the coexistence between technology and caring.

Five assumptions support this theory: First, *Persons are caring by virtue of their humanness*. In interpreting this assumption, it allows for the realization that while all persons are caring, their expressions of caring are different. This realization is critical to appreciating the idea of persons as being caring. Second, *The ideal of wholeness is a perspective of oneness*. “The conceptualization of wholeness allows for the acknowledgement of human beings as complete-in their-being, without reference to composition of parts” (Locsin & Purnell, 2015). This ideal allows the nurse to focus on nursing as a shared lived experience between the nurse and their patients (Boykin & Schoenhofer, 2001) rather than on fixing the person or completing the person’s deficiency or missing parts.

Third, *Knowing persons as caring is a multidimensional process* (Locsin, 2005) in which the nurse and their patients focus on appreciating, celebrating, supporting, and affirming each other while allowing for mutual recognition as dynamic participants in human caring. The fourth assumption is that *technologies of health and nursing are elements for caring* (Locsin, 2005). Through these technologies nurses in practice are able to know their patients more fully as persons, who are active contributors in their care rather than simply being objects of care. And last, *Nursing is a discipline of knowledge and a practice profession*. As a discipline and a profession, knowledge development is paramount to its existence. Advances in nursing and its practices are focused on enhancing its legitimate contribution in health care, in which its practice is geared towards the attainment and maintenance of human health and well-being.

## Why Should Nursing Drive Technological Developments for Human Caring?

As nursing has become more professionalized, nurses have gained recognition as well educated healthcare providers. They are seen as healthcare providers who are capable of great autonomy of both action and thought. Nurses should use their individual and collective voices to push for changes that expand nurse autonomy and increase the reach and influence of their profession. In an age driven by technological developments, nurses must take an active role in the development of technology in healthcare. The Biodesign process necessitates input from frontline nurses and other multidisciplinary healthcare team members for the development of technologies catering to patient needs while ensuring delivery of a more holistic type of care.

As nursing is the expression of caring (Anderson, Wilaman, Strand, & Borglin, 2015), the design of technologies used in healthcare should be based on “Person-Centered Care” (Zhao, Gao, Wang, Liu, & Hao, 2016) focused on an individual’s desires, needs, wants, and goals that are central to nursing care. Practicing human caring indicates that there is a strong interest in the experience of patients on health, injury, illness, needs, freedom, etc. Patient Centered Care is a holistic approach to delivering care that is respectful and individualized, allowing negotiations of care, and offering choice through therapeutic relationship where persons are empowered to be involved in health decisions (Locsin & Purnell, 2015; Poey et al., 2017).

## Knowing Persons as Caring: A Process of Nursing

The process of nursing is to be considered as “knowing persons as caring” (Locsin, 2005). This process of nursing can be used as the impetus to drive technological developments in healthcare thereby generating purposeful health care technologies. The design, development, and use of technologies for human caring are for the purpose of facilitating knowing persons as caring. Using the theory of Technological Competency as Caring in Nursing as a guide for developing technologies for knowing persons as caring ensures that these technological developments are responsive to the societal demands of caring in to-day’s context.

Knowing persons as caring’ is the process of nursing that unfolds as* technological knowing, mutual designing, and participative engaging*. These three processes of nursing practice are grounded in the theory of Technological Competency as Caring in Nursing (Locsin, 2016). Technological knowing is the use of technologies in order to know persons more fully as caring persons. With technological knowing, the advantage of knowing technologies and having technological expertise are greatly appreciated. Having these information and skills allows nursing engagement between the nurse and nursed. Mutual designing underscores the understanding of human beings as persons who are participants in the care rather than simply comprised of biological organs.

Nurses can promote the use of technology requiring a system recognizing the needs of patients. The most important thing to consider is to ensure that the human element of care is not lost in the expansion of technology (Hamer, 2013). Nurses involved in the development of technology are integral elements of technological development in healthcare because they are the deciding factor in the selection, implementation, and evaluation of technologies in healthcare that support safe, high-quality, and patient-centered care. Nursing is finding meaning between achieving the benefits of using technology while not depreciating the human element (Cassano, 2014; Elkind, 2009).

## Nursing Implications

While nurses should be actively involved in the development of technologies for patient care, some-times, nurses cannot forcibly ensure that the technologies being developed enable them to practice human caring within a human caring perspective. Because nurses are forefront practitioners in healthcare, nursing involvement in technological development is to ensure that the technology being developed is relevant to local health care conditions in which assimilation, adaptation, and adoption are made seamless especially in developing countries in the Asia-Pacific region.

## Conclusion

How can nursing and its practice drive technological advancement in human caring? As a quintessential question, the answer can provide a critical avenue for contemporary and future nursing engagements in human caring central to advancing technological competencies as expressions of caring in nursing. One of the most important responsibilities of nurses is involvement in technological innovations, safeguarding the vital element of humanness in the development of technologies that drive human health care.

## Declaration of Conflicting Interests

The authors declared no potential conflicts of interest concerning the research, authorship, or publication of this article.
